# Durable Suppression of HIV-1 after Virologic Monitoring-Based Antiretroviral Adherence Counseling in Rakai, Uganda

**DOI:** 10.1371/journal.pone.0127235

**Published:** 2015-05-26

**Authors:** Alexander Billioux, Gertrude Nakigozi, Kevin Newell, Larry W. Chang, Thomas C. Quinn, Ron H. Gray, Anthony Ndyanabo, Ronald Galiwango, Valerian Kiggundu, David Serwadda, Steven J Reynolds

**Affiliations:** 1 Johns Hopkins School of Medicine, Baltimore, Maryland; 2 Rakai Health Sciences Program, Rakai, Uganda; 3 Clinical Research Directorate/Clinical Monitoring Research Program, Leidos Biomedical Research, Inc., Frederick National Laboratory for Cancer Research, Frederick, Maryland; 4 National Institute for Allergy and Infectious Diseases, National Institutes of Health, Bethesda, Maryland; 5 Johns Hopkins Bloomberg School of Public Health, Baltimore, Maryland; 6 Makerere University College of Health Sciences, School of Public Health, Kampala, Uganda; University of British Columbia, CANADA

## Abstract

**Objectives:**

HIV viral load is recommended for monitoring antiretroviral treatment and identifying treatment failure. We assessed the durability of viral suppression after viral load-triggered adherence counseling among patients with HIV viremia 6 months after ART initiation.

**Design:**

Observational cohort enrolled in an antiretroviral treatment program in rural Uganda.

**Methods:**

Participants who underwent routine viral load determination every 24 weeks and had at least 48 weeks of follow-up were included in this analysis. Patients with viral loads >400 copies/ml at 24 weeks of treatment were given additional adherence counseling, and all patients were followed to assess the duration of viral suppression and development of virologic failure.

**Results:**

1,841 participants initiating antiretroviral therapy were enrolled in the Rakai Health Sciences Program between June 2005 and June 2011 and were followed with viral load monitoring every 24 weeks. 148 (8%) of patients did not achieve viral suppression at 24 weeks and were given additional adherence counseling. 85 (60%) of these patients had undetectable viral loads at 48 weeks, with a median duration of viral suppression of 240 weeks (IQR 193-288 weeks). Failure to achieve an undetectable viral load at 48 weeks was associated with age <30 years and 24 week viral load >2,000 copies/ml in multivariate logistic regression analysis.

**Conclusions:**

The majority of patients with persistent viremia who were provided adherence counseling achieved robust viral suppression for a median 4.6 years. Access to virologic monitoring and adherence counseling is a priority in resource-limited settings.

## Introduction

Adherence to antiretroviral therapy (ART) is critical for successful treatment of HIV infection. Consistent ART use leads to viral suppression and dramatically reduced morbidity and mortality [1,2]. Additionally, virologic suppression reduces HIV transmission to the partners of infected individuals and can decrease incidence within communities [3,4]. However, achieving these benefits requires high levels of treatment adherence—generally estimated to be 90–95%—which many patients find difficult to maintain [5–8]. Adherence may be improved through motivational patient counseling, involvement of peer treatment support, and text message reminder systems, but the resource requirements of these interventions may limit their implementation as the standard of care in large, resource-constrained programs [9–11]. Therefore, identifying patients at higher risk of poor adherence and subsequent treatment failure is a priority.

The best indicator of adherence and response to treatment is the virologic response [12]. Accordingly, the World Health Organization (WHO) and many national AIDS control programs have adopted guidelines recommending viral load testing 3 to 6 months after initiating ART and then at regular intervals thereafter [13]. While these viral load determinations are primarily performed to identify early treatment failure, recent studies have demonstrated that virologic monitoring may also help identify individuals with slow response to therapy who might benefit from early adherence interventions to avoid treatment failure [14]. Between 57–84% of patients in these studies achieve viral suppression or re-suppression after targeted adherence interventions; however, few studies have reported maintenance of suppression beyond 1 year of follow-up. The objective of this study was to determine whether patients in ART clinics in rural Rakai District, Uganda with slow initial virologic response to therapy maintained long-term viral suppression after targeted adherence interventions.

## Methods

Rakai District, located in rural southwestern Uganda, has one of the highest HIV prevalences in Uganda. The Rakai Health Sciences Program (RHSP), funded by the President’s Emergency Plan for AIDS Relief (PEPFAR), has provided free antiretroviral therapy (ART) since June, 2004 through mobile outreach clinics with biweekly visits to 16 regional health clinics. Starting in 2005, viral load monitoring was introduced to follow all patients on ART. Between June 2005 and June 2011, 2,365 ART-naïve adult (age 18 years or more) participants were enrolled in an open cohort and after ART initiation based on a CD4 cell count <250 cells/mm^3^ or WHO stage IV disease. Initial treatment regimens consisted of two NRTIs (zidovudine or stavudine plus lamivudine) and nevirapine or efavirenz. Participants were seen weekly for the first month and then biweekly for 2 months followed by monthly follow-up appointments with adherence and HIV risk reduction counseling at all visits. HIV-1 viral load testing using the Roche Amplicor 1.5 Monitor assay (Roche Diagnostics, Indiana, USA) was used to monitor all ART clients every 24 weeks. Individualized adherence plans were developed for patients with a viral load >400 copies/μl at 24 weeks by a multi-disciplinary team composed of physicians, clinic officers, and counselors, with interventions including additional adherence counseling in a one-on-one session with clinicians, assignment of a peer treatment supporter, or in-depth psychosocial counseling interventions by specialized staff.

Baseline characteristics were compared among early suppressors, late suppressors, and non-suppressors using chi-square tests for categorical variables and analysis of variance for continuous variables. We assessed predictors of non-suppression by week 48 among n = 142 patients who failed to suppress by week 24 using stepwise logistic regression to estimate odds ratios associated with gender, age, baseline CD4 cell count, baseline WHO stage and 24 week viral load. Stepwise regression entry criterion was set as p = 0.3 and stay criterion was set as p = 0.15. In order to calculate duration of virolgic suppression, we censored subjects at the time of their last available viral load result. All analyses were conducted using SAS version 9.2.

The study was approved by the Uganda Virus Research Institute Scientific and Ethics Committee, The Johns Hopkins University IRB and the Uganda National Council for Science and Technology.

## Results

**Fig 1:**
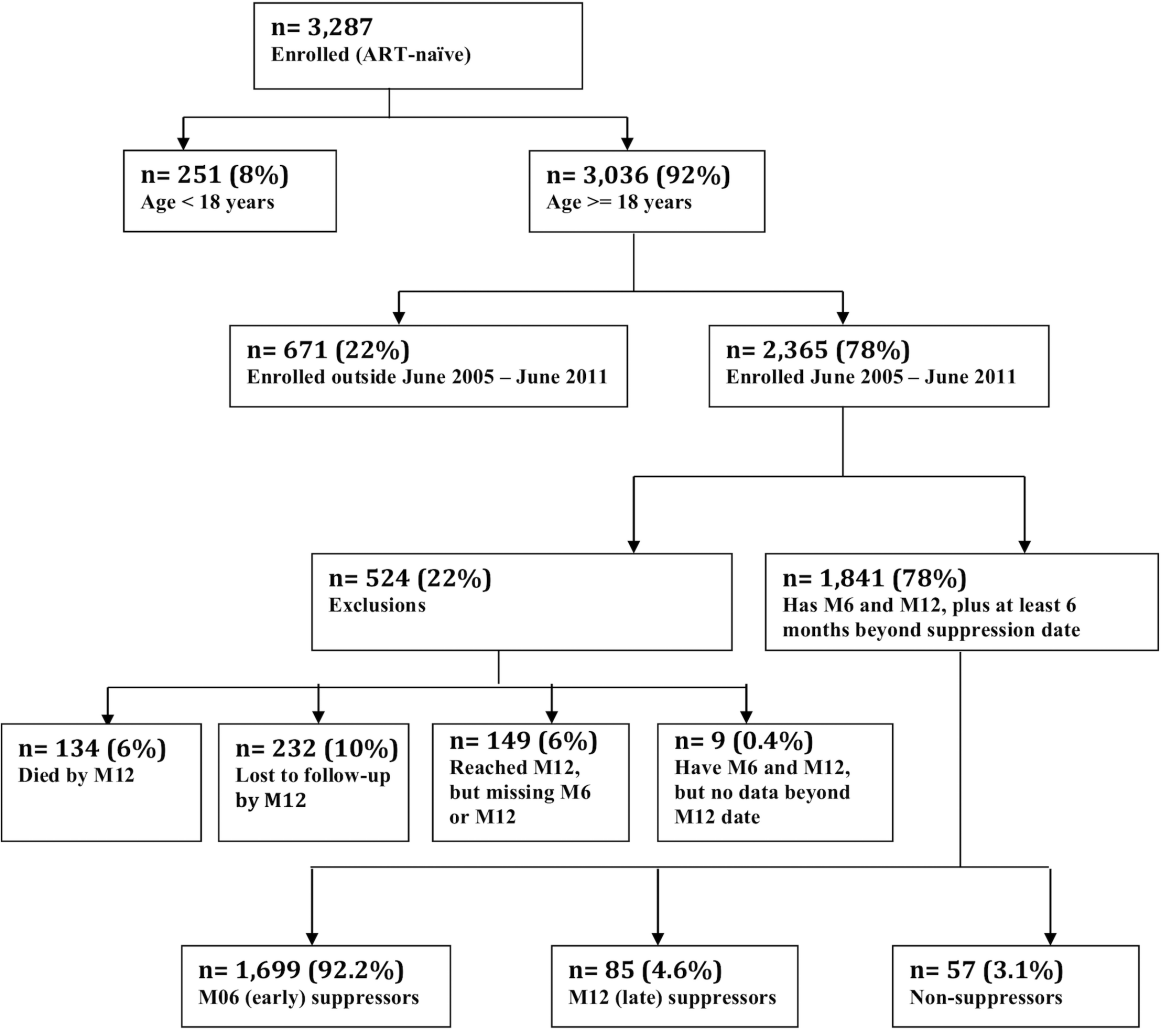
RHSP ART Program Enrollment and Selection of Analysis Participants.

Between June, 2005 and June, 2011, 2,365 ART-naïve patients were initiated on ART (Fig 1). We included the 1,841 (77.8%) patients alive and in care at least 48 weeks after initiation in this analysis. Baseline characteristics according to virologic outcome categories are presented in Table 1. To summarize, median age among these patients was 33 years (IQR = 28–40) and 1220 (66.3%) were female. The proportion of patients initiated on a d4T-containing regimen was 19%, while 80% were initiated on a regimen containing 3TC/AZT. Median (IQR) for baseline CD4+ count was 186 (107–226), and for baseline HIV-1 viral load was 73426 (17417–234728). Thirty-eight (38%) of patients were WHO stage I at baseline, 37% stage II, 18% stage III and 6% stage IV. The overall prevalence of virologic failure at week 24 for the entire cohort was 9%. Of the 1,841 patients in care at 48 weeks included in this analysis, 1,699 (92%) had a 24 week viral load <400 copies/ml and were termed ‘early suppressors.’ The remaining 142 patients had a median viral load of 1020 copies/ml (IQR 543–2,974 copies/ml) at 24 weeks. These patients underwent additional adherence counseling and were continued on first-line therapy. At 48 weeks, 85 (60%) of these patients achieved a viral load <400 copies/ml and were termed ‘late suppressors.’ Fifty-seven (3%) patients included in this analysis never achieved viral suppression with first-line regimens and were termed “non-suppressors.”

**Table 1:** Baseline characteristics and analysis of the study population.

**Baseline characteristics of the study population**				
**Characteristic**	**Early Suppressors** N = 1699 (92%)	**Late Suppressors** N = 85 (5%)	**Non-Suppressor** N = 57 (3%)	**P-value**
Age Median (IQR)	33 (28–40)	34 (30–41)	30 (26–35)	0.013
**Gender**				0.383
Female	1132 (67%)	55 (65%)	33 (58%)	
Male	567 (33%)	30 (35%)	24 (42%)	
Baseline CD4 cells/ul Median (IQR)	188 (108–227)	162 (106–214)	146 (61–215)	0.020
Baseline VL copies/ml Median (IQR)	72324 (16319–223996)	103426 (27443–348847)	77033 (28503–297333)	0.156
**WHO Stage**				0.006
Stage I	659 (39%)	19 (22%)	21 (37%)	
Stage II	629 (37%)	34 (40%)	20 (35%)	
Stage III	304 (18%)	26 (31%)	8 (14%)	
Stage IV	99 (6%)	6 (7%)	8 (14%)	
**ART Regimen**				0.527
d4T/3TC/NVP	206 (12%)	15 (18%)	9 (16%)	
d4T/3TC/EFV	100 (6%)	12 (14%)	2 (4%)	
CBV/NVP	919 (54%)	37 (44%)	30 (54%)	
CBV/EFV	446 (26%)	21 (25%)	14 (25%)	
Other	27 (1%)	0	1 (2%)	
**Univariate Odds Ratio Estimates for Non-Suppression at 48 Weeks**				
		**Univariate OR**	**95% CI**	**p-value**
Age < 30 years		2.9	1.4–6.0	0.0036
Gender (F vs M)		0.75	0.38–1.5	0.4130
Week 24 viral load > 2000 copies/ml		7.7	3.6–16.6	<0.0001
CD4 < 100 at BL		1.9	0.87–4.0	0.1081
WHO Stage 3 or 4 at BL (vs Stage 1 or 2)		1.5	0.75–3.2	0.2384
**Adjusted Odds Ratio Estimates for Non-Suppression at 48 Weeks**				
		**Adjusted OR**	**95% CI**	**p-value**
Age < 30 years		2.7	1.2–6.1	0.0163
Week 24 viral load > 2000 copies/ml		7.4	3.4–16.3	<0.0001

Early suppressors, late suppressors, and non-suppressors differed significantly by age, baseline CD4 count, and WHO clinical stage at time of enrollment (Table 1), with early suppressors having a higher median CD4 count (188 copies/μl) and lower percentage of patients with stage IV disease (6%). There was no difference in gender, baseline viral load, or first-line ART regimen. Failure to achieve an undetectable viral load at 48 weeks was associated with age <30 years and 24 week viral load >2,000 copies/ml in multivariate logistic regression analysis (Table 1).

The majority of late suppressors, 71/85 (84%), remained suppressed well beyond 48 weeks, with median sustained suppression of 4.6 years (IQR 3.7–5.5 years). Virologic failure was identified in 193 (10%) patients by 48 weeks of treatment, including 179/1699 (11%) of early suppressors and 14/84 (16%) of late suppressors. There was no significant difference in the median time to virologic failure between these two groups (72 weeks versus 86 weeks, p = 0.85).

## Discussion

Viral load monitoring remains a critical component of HIV care, to evaluate treatment efficacy and provide early warnings of resistance and treatment failure [15]. This study demonstrates that early determination of viral load may help avoid downstream virologic failure with targeted adherence counseling. While the majority of patients in our cohort achieved suppressed viral loads by 24 weeks of treatment, a subset of patients with persistent viremia at this time point were provided further adherence support. Subsequently, the majority of these patients achieved viral suppression when retested 24 weeks later. These late suppressors were subsequently found to maintain suppressed viral loads for a median of 4.6 years, one of the longest follow-up terms reported in the literature. It is important to note that our findings may overestimate the effectiveness the counseling intervention, as those with virologic failure whom remained non-adherent to their medication may have been at greater risk of dying before reaching one year on treatment.

Our findings add to evidence supporting the importance of early viral load determination after ART initiation. In addition to allowing for earlier diagnosis of virologic failure [16], others have found viral load testing 6 months after ART initiation to be predictive of future virologic failure [12], HIV disease progression [17], and mortality [18,19]. Programs that employ viral load testing are also more likely to detect treatment failure earlier in therapy than programs relying on immunological or clinical monitoring, though no difference in mortality has been demonstrated between patients managed using CD4 counts or viral loads to determine treatment failure [20–23]. Reducing the duration of HIV viremia decreases the likelihood of developing viral resistance and achieving viral suppression [24,25].

The current study reinforces the utility of viral load testing to identify patients with persistent viremia who may benefit from further adherence counseling in order to avoid treatment failure. A recent systematic review found that programs employing such viral load-triggered adherence interventions were able to achieve substantial rates of viral suppression or re-suppression, though the percentage of patients achieving suppression varied considerably between studies [14]. A study from Thailand reported results similar to ours, with 92% of patients with persistent low-level viremia achieving viral suppression after additional adherence counseling [26]. However. Studies in Burkina Faso and Mali found that only one-third of patients receiving viral-load triggered adherence interventions were able to achieve undetectable viremia [27]. However, patients in these cohorts had been treated with ART for a mean of 23.7 months prior to their first viral load determination and were found to have high rates of resistance mutations. All patients in our cohort were monitored virologically from 6 months after ART initiation, and virologic failure rates were consistent with median values reported by other programs in resource-limited settings [28]. Notably, the longest period of follow-up after viral suppression reported in the review was 18 months [29]. Therefore our median duration of suppression after viral load-triggered adherence counseling of 4.6 years is unique and supports the value of early viral load monitoring in resource-limited settings.

This study’s retrospective design precludes strong causal inferences about the impact of adherence counseling on viral suppression in patients with persistent viremia. Indeed the high rates of initial suppression observed, as well as the success of our targeted adherence interventions, may in part reflect the effects of closely monitoring viral load itself, rather than the added counseling. Nevertheless, given the growing number of studies reporting an association between viral load-triggered adherence counseling and improved rates of subsequent suppression adds plausibility to the benefits of this intervention. Our findings from a large cohort of patients receiving ART support early viral load monitoring and targeted adherence programs in resource-limited settings.

## References

[1] Mannheimer S, Friedland G, Matts J, Child C, Chesney M (2002) The Consistency of Adherence to Antiretroviral Therapy Predicts Biologic Outcomes for Human Immunodeficiency Virus-Infected Persons in Clinical Trials. 34: 1115–1121. 10.2307/4483031?ref=search-gateway:d9cad5bafdaa766de511aa669f0b877e 11915001

[2] McNabb J, Ross JW, Abriola K, Turley C, Nightingale CH, Nicolau DP. (2001) Adherence to Highly Active Antiretroviral Therapy Predicts Virologic Outcome at an Inner-City Human Immunodeficiency Virus Clinic. CLIN INFECT DIS 33: 700–705. 10.2307/4461667?ref=search-gateway:612983b90198260ad7ee263bc6ec2bea 11486292

[3] Cohen MS, Chen YQ, McCauley M, Gamble T, Hosseinipour MC, Kumarasamy N, et al (2011) Prevention of HIV-1 Infection with Early Antiretroviral Therapy—NEJM. N Engl J Med 365: 493–505. Available: http://www.nejm.org/doi/full/10.1056/nejmoa1105243. 10.1056/NEJMoa1105243 21767103 PMC3200068

[4] Tanser F, Bärnighausen T, Grapsa E, Zaidi J, Newell M-L (2013) High Coverage of ART Associated with Decline in Risk of HIV Acquisition in Rural KwaZulu-Natal, South Africa. Science 339: 966–971. 10.1126/science.1228160 23430656 PMC4255272

[5] Paterson DL, Swindells S, Mohr J, Brester M, Vergis EN, Squier C, et al (2000) Adherence to Protease Inhibitor Therapy and Outcomes in Patients with HIV Infection. Ann Intern Med 133: 21–30. 10.7326/0003-4819-133-1-200007040-00004 10877736

[6] Howard AA, Arnsten JH, Lo Y, Vlahov D, Rich JD, Schuman P, et al (2002) A prospective study of adherence and viral load in a large multi-center cohort of HIV-infected women. AIDS 16: 2175–2182. 12409739 10.1097/00002030-200211080-00010

[7] von Wyl V, Klimkait T, Yerly S, Nicca D, Furrer H, Cavassini M, et al (2013) Adherence as a Predictor of the Development of Class-Specific Resistance Mutations: The Swiss HIV Cohort Study. PLoS ONE 8: e77691 10.1371/journal.pone.0077691 24147057 PMC3797701

[8] Mills EJ, Nachega JB, Buchan I, Orbinski J, Attaran A, Singh S, et al (2006) Adherence to Antiretroviral Therapy in Sub-Saharan Africa and North America: A Meta-analysis. JAMA 296: 679–690. 10.1001/jama.296.6.679 16896111

[9] Enriquez M, McKinsey (2011) Strategies to improve HIV treatment adherence in developed countries: clinical management at the individual level. HIV: 45. 10.2147/HIV.S8993 PMC3218706 22096406

[10] Medley A, Kennedy C, O'Reilly K, Sweat M (2009) Effectiveness of peer education interventions for HIV prevention in developing countries: a systematic review and meta-analysis. AIDS Educ Prev 21: 181–206. 10.1521/aeap.2009.21.3.181 19519235 PMC3927325

[11] Chang LW, Kagaayi J, Nakigozi G, Ssempijja V, Packer AH, Serwadda D, et al (2010) Effect of peer health workers on AIDS care in Rakai, Uganda: a cluster-randomized trial. PLoS ONE 5: e10923 10.1371/journal.pone.0010923 20532194 PMC2880005

[12] Almeida JM, Letang E, Nhampossa T, Ayala E, David C, Menendez C, et al (2011) Rapid Suppression of HIV-RNA Is Associated with Improved Control of Immune Activation in Mozambican Adults Initiating Antiretroviral Therapy with Low CD4 Counts. AIDS Research and Human Retroviruses 27: 705–711. 10.1089/aid.2010.0200 21091388

[13] World Health Organization (2013) Consolidated Guidelines on the use of Antiretroviral Drugs for Treating and Preventing HIV Infection. 272 pp. 24716260

[14] Bonner K, Mezochow A, Roberts T, Ford N, Cohn J (2013) Viral load monitoring as a tool to reinforce adherence: a systematic review. J Acquir Immune Defic Syndr 64: 74–78. 10.1097/QAI.0b013e31829f05ac 23774877

[15] Keiser O, Chi BH, Gsponer T, Boulle A, Orrell C, Phiri S, et al (2011) Outcomes of antiretroviral treatment in programmes with and without routine viral load monitoring in Southern Africa. AIDS 25: 1761–1769. 10.1097/QAD.0b013e328349822f 21681057 PMC3605707

[16] Alvarez-Uria G, Naik PK, Pakam R, Midde M (2012) Early HIV viral load determination after initiating first-line antiretroviral therapy for indentifying patients with high risk of developing virological failure: data from a cohort study in a resource-limited setting. Trop Med Int Health 17: 1152–1155. 10.1111/j.1365-3156.2012.02982.x 22487689

[17] Grabar S, Moing VL, Goujard C, Egger M, Leport C, Kazatchkine MD, et al (2005) Response to Highly Active Antiretroviral Therapy at 6 Months and Long-Term Disease Progression in HIV-1 Infection. JAIDS Journal of Acquired Immune Deficiency Syndromes 39: 284 15980687 10.1097/01.qai.0000160925.33935.72

[18] De Beaudrap P, Etard J-F, Ecochard R, Diouf A, Dieng AB, Cilote V, et al (2008) Change over time of mortality predictors after HAART initiation in a Senegalese cohort. Eur J Epidemiol 23: 227–234. 10.1007/s10654-007-9221-3 18197359

[19] Fregonese F, Collins IJ, Jourdain G, Lecoeur S, Cressey TR, Ngo-Giang-Houng N, et al (2012) Predictors of 5-year mortality in HIV-infected adults starting highly active antiretroviral therapy in Thailand. J Acquir Immune Defic Syndr 60: 91–98. 10.1097/QAI.0b013e31824bd33f 22293548

[20] ART-LINC of IeDEA Study Group, Keiser O, Tweya H, Boulle A, Braitstein P, Schecter M, et al (2009) Switching to second-line antiretroviral therapy in resource-limited settings: comparison of programmes with and without viral load monitoring. AIDS 23: 1867–1874. 10.1097/QAD.0b013e32832e05b2 19531928 PMC2956749

[21] Reynolds SJ, Nakigozi G, Newell K, Ndyanabo A, Galiwongo R, Boaz I, et al (2009) Failure of immunologic criteria to appropriately identify antiretroviral treatment failure in Uganda. AIDS 23: 697–700. 10.1097/QAD.0b013e3283262a78 19209067 PMC2720562

[22] Mermin J, Ekwaru JP, Were W, Degerman R, Bunnell R, Kaharuza F, et al (2011) Utility of routine viral load, CD4 cell count, and clinical monitoring among adults with HIV receiving antiretroviral therapy in Uganda: randomised trial. BMJ 343: d6792–d6792. 10.1136/bmj.d6792 22074711 PMC3213241

[23] Jourdain G, Le Cœur S, Ngo-Giang-Huong N, Traisathit P, Cressey TR, Fregonese F, et al (2013) Switching HIV treatment in adults based on CD4 count versus viral load monitoring: a randomized, non-inferiority trial in Thailand. PLoS Med 10: e1001494 10.1371/journal.pmed.1001494 23940461 PMC3735458

[24] Townsend D, Troya J, Maida I, Pérez-Saleme L, Satta G, Wilkin A, et al (2009) First HAART in HIV-infected patients with high viral load: value of HIV RNA levels at 12 weeks to predict virologic outcome. J Int Assoc Physicians AIDS Care (Chic) 8: 314–317. 10.1177/1545109709343966 19759257

[25] Metzner KJ, Allers K, Rauch P, Harrer T (2007) Rapid selection of drug-resistant HIV-1 during the first months of suppressive ART in treatment-naive patients. AIDS 21: 703–711. 10.1097/QAD.0b013e3280121ac6 17413691

[26] Wilson D, Keiluhu AK, Kogrum S, Reid T, Seriratana N, Ford N, et al (2009) HIV-1 viral load monitoring: an opportunity to reinforce treatment adherence in a resource-limited setting in Thailand. Transactions of the Royal Society of Tropical Medicine and Hygiene 103: 601–606. 10.1016/j.trstmh.2008.11.007 19110288

[27] Pirkle CM, Boileau C, Nguyen VK, Machouf N, Ag Aboubacrine S, Niamba PA, et al (2009) Impact of a modified directly administered antiretroviral treatment intervention on virological outcome in HIV‐infected patients treated in Burkina Faso and Mali. HIV Med 10: 152–156. 10.1111/j.1468-1293.2008.00664.x 19245536

[28] Aghokeng AF, Monleau M, Eymard-Duvernay S, Dagnra A, Kania D, Ngo-Giang-Huong N, et al (2014) Extraordinary heterogeneity of virological outcomes in patients receiving highly antiretroviral therapy and monitored with the World Health Organization public health approach in sub-saharan Africa and southeast Asia. CLIN INFECT DIS 58: 99–109. 10.1093/cid/cit627 24076968

[29] Coetzee D, Boulle A, Hildebrand K, Asselman V, van Cutsem G, Goemaere E (2004) Promoting adherence to antiretroviral therapy: the experience from a primary care setting in Khayelitsha, South Africa. AIDS 18 Suppl 3: S27–S31. 10.1097/01.aids.0000131322.13515.e2 15322481

